# ﻿A redescription and two new descriptions of gnathiid isopods (Isopoda, Gnathiidae) from South African museum collections (1898–1976)

**DOI:** 10.3897/zookeys.1256.162445

**Published:** 2025-10-17

**Authors:** Hesmarié Botha, Nico J. Smit, Anja Erasmus, Kerry A. Hadfield

**Affiliations:** 1 Water Research Group, Unit for Environmental Sciences and Management, North-West University, Private Bag X6001, Potchefstroom, 2520, South Africa North-West University Potchefstroom South Africa; 2 South African Institute for Aquatic Biodiversity, Private Bag 1015, Makhanda 6140, South Africa South African Institute for Aquatic Biodiversity Makhanda South Africa

**Keywords:** *
Gnathia
*, life below water, morphology, taxonomy, temporary parasite

## Abstract

Museum collections continue to play a critical role in taxonomic research by preserving historical material and biodiversity records that might otherwise have been lost. In the present study, material from the Iziko South African Museum, Cape Town, was used to morphologically redescribe *Gnathia spongicola* Barnard, 1920, as well as describe two new species, *Gnathia brevicula***sp. nov.** and *Gnathia lancifera***sp. nov.**, from the Temperate Southern Africa (TSA) marine realm. The triangular anterior margin of pereonite 6, with its midpoint projecting forward to reach pereonite 4 and bisect pereonite 5, is a feature rarely recorded among Gnathiidae (previously noted only in *Gnathia disjuncta*). However, this character is present in all three species examined in the present study. Of the three, *Gnathia spongicola* is characterised by a weak and bifid mediofrontal process; a single superior frontolateral process; strong and equally apically bifid, pronounced and pointed supraocular lobes; as well as strong distally curved mandibles with dentated blades. *Gnathia brevicula***sp. nov.** is distinguished by two strong, rounded and extended frontolateral processes; broadly rounded and minimally developed supraocular lobes; and weakly curved mandibles with slight distal curvature. *Gnathia lancifera***sp. nov.** can be characterised by a mediofrontal process that is weakly rounded; two strong and conical superior frontolateral processes; rounded and pronounced supraocular lobes; and strong, distally curved, crescent-shaped mandibles with dentation. These descriptions increase the number of known species of Gnathiidae from the TSA marine realm to 11.

## ﻿Introduction

Historical natural history collections housed in museums around the world have often provided the opportunity for researchers to revise taxonomic groups and describe new species from ancient material. This is particularly true for fish-parasitic isopods in the families Cymothoidae and Gnathiidae. In the case of the permanently attached cymothoids, specimens were typically obtained from hosts captured in trawls and nets (Bruce 1986, 1987; Hadfield et al. 2016), while for temporarily parasitic gnathiids, free-living adult stages were collected during large-scale benthic surveys and subsequently deposited in museum collections (Cohen and Poore 1994; Smit and Davies 2004 and references therein). In South Africa, gnathiid isopods deposited in the Iziko South African Museum (SAM), Cape Town, during the 20^th^ century have proven invaluable in enhancing our understanding of gnathiid taxonomy and diversity. For example, Smit et al. (2000) redescribed and reassigned *Gnathia cryptopais* Barnard, 1925 to *Caecognathia cryptopais* (Barnard, 1925) based on specimens collected in 1902; Smit and Van As (2000) described *Gnathia nkulu* Smit & Van As, 2000 from males collected between 1972 and 1978; and Hadfield and Smit (2008) described the new genus and species *Afrignathia multicavea* Hadfield & Smit, 2008 from material collected between 1961 and 1972, all of which were based on specimens housed at SAM.

**Figure 1.**
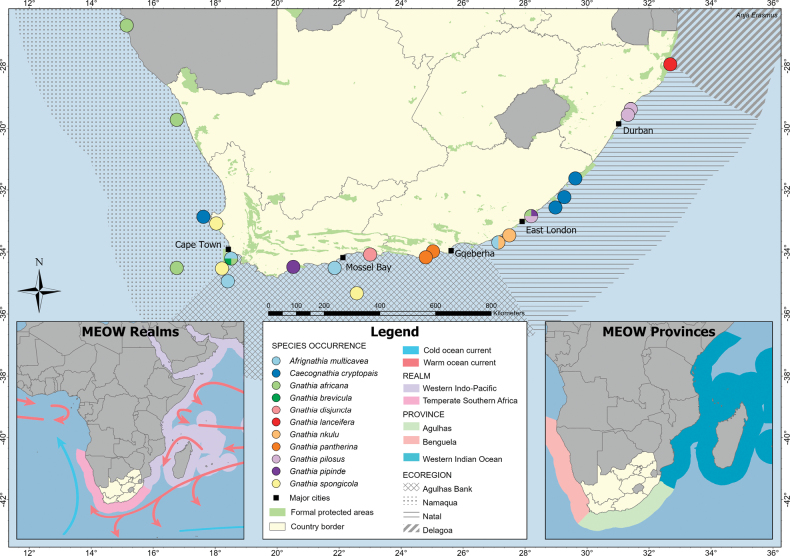
Map showing the known distribution of the Gnathiidae species from the Temperate Southern African (TSA) and Western Indo-Pacific (WIP) marine realms. The associated MEOW (Marine Ecoregions of the World) realms, provinces, and ecoregions are illustrated, based on Spalding et al. (2007).

In the present study, historical material from the SAM collections was again used to further our knowledge of gnathiid isopods from the Temperate Southern Africa (TSA) marine realm. Defined by Spalding et al. (2007) in the Marine Ecoregions of the World (MEOW) classification, this realm includes the temperate coastal and shelf waters of South Africa and Namibia. As part of this work, a comprehensive redescription of *Gnathia spongicola* Barnard, 1920 is provided, based on the original type material. Additionally, examination of all SAM specimens labelled as *G. spongicola* revealed two distinct species of *Gnathia* Leach, 1814 that do not conform to the morphology of *G. spongicola* or any other known gnathiid species. These are described here as new to science. The addition of these two species increases the number of known TSA gnathiid species to 11, across three genera (Table 1), and extends the documented distribution of gnathiids in this region (Fig. 1).

**Table 1.** Species list of the Gnathiidae from the Temperate Southern Africa (TSA) region, including information on distribution, depth, size, substrate, host families, and life stage.

Species	Province and ecoregion	Depth (m)	Size (mm)	Substratum/host family	Life stage described (M/F/J)*	References
*Afrignathia multicavea* Hadfield & Smit, 2008	Agulhas (Agulhas Bank and Natal); Benguela (Namaqua)	26–73	1.5–2.0	Unknown	M	Hadfield and Smit (2008)
*Caecognathia cryptopais* (Barnard, 1925)	Agulhas (Natal)	160–775 **	2.0–3.9	Unknown	M	Barnard (1925); Smit et al. (2000)
*Gnathia africana* Barnard, 1914	Agulhas (Agulhas Bank and Natal); Benguela (Namaqua)	Intertidal	3.7–5.1	Clinidae Swainson, 1839; Gobiesocidae Bleeker, 1859; Gobiidae Cuvier, 1816; Halichondriidae Gray, 1867; Polymastiidae Gray, 1867	M/F/J	Barnard (1914); Smit and Davies (1999); Smit et al. (1999); Erasmus et al. (2022)
***Gnathia brevicula* sp. nov.**	Agulhas (Agulhas Bank)	55–77	2.3–2.5	Branching sponges	M	Barnard (1920); current study
*Gnathia disjuncta* Barnard, 1920	Agulhas (Agulhas Bank)	73	3.5	Unknown	M	Barnard (1920)
***Gnathia lancifera* sp. nov.**	Agulhas (Natal)	550	3.1	Unknown	M	Current study
*Gnathia nkulu* Smit & Van As, 2000	Agulhas (Agulhas Bank and Natal) ***	80–200	3.3–4.9	Unknown	M	Smit and Van As (2000); Kensley et al. (2009)
*Gnathia pantherina* Smit & Basson, 2002	Agulhas (Agulhas Bank)	Intertidal	3.7–6.8	Scyliorhinidae Gill, 1862; Torpedinidae Henle, 1834	M/F/J	Smit and Basson (2002)
*Gnathia pilosus* Hadfield, Smit & Avenant-Oldewage, 2008	Agulhas (Natal)	Intertidal	1.6–2	Acanthuridae Bonaparte, 1835; Blenniidae Rafinesque, 1810; Epinephelidae Bleeker, 1874; Gobiidae Cuvier, 1816; Labridae Cuvier, 1816; Pomacentridae Bonaparte, 1831; Scorpaenidae Risso, 1827; Sparidae Rafinesque, 1818; Terapontidae Richardson, 1842	M/F/J	Hadfield et al. (2009); Erasmus et al. (2022)
*Gnathia pipinde* Smit & Hadfield, 2022	Agulhas (Agulhas Bank and Natal)	Intertidal-shallow; subtidal	3.8–4.6	Tetraodontidae Bonaparte, 1831	M/J	Smit and Hadfield (2022)
***Gnathia spongicola* Barnard, 1920**	Agulhas (Agulhas Bank); Benguela (Namaqua)	200–347	3.5–4.7	Hexactinellida sponges	M	Barnard (1920); current study

*Life stage described. M = male; F = female, J = juvenile. **Additional depths acquired from available museum data. ***Gnathia nkulu was also recorded off the coast of Madagascar (Kensley et al. 2009), in the Western Indo-Pacific (WIP) realm.

## ﻿Materials and methods

This study makes use of material housed in the Iziko South African Museum, collected between 1898 and 1976. Ethical clearance for this study was obtained through the North-West University AnimCare animal ethics committee (NWU-01264-24-A9 and NWU-00784-24-A5).

A total of 22 males of *Gnathia spongicola* and 301 males previously referred to by Barnard (1920) as G. spongicola var. minor were collected off the coast of the Western Cape province, from both the Agulhas Bank and Namaqua ecoregions. Additionally, three males of another undescribed species were collected off the coast of KwaZulu-Natal province, within the Natal ecoregion (Fig. 1). All females and juveniles were excluded, as there is no clear evidence that they belong to the same species as the males.

For scanning electron microscopy (SEM), specimens were rehydrated from 70% ethanol to fresh water, where they were rinsed for 24–36 hours, depending on the level of surface contamination, to remove any remaining debris. Subsequent dehydration was carried out using a graded ethanol series, followed by critical point drying using standard procedures (Smit et al. 1999). Dried specimens were mounted on conical stubs using the rapid drying varnish, Japan Gold Size (Winsor and Newton), sputter-coated with gold, and examined under a JEOL JSM 6400 scanning electron microscope at 10 kV, with the stage angled between 70° and 90°.

Light microscopy was performed using Nikon Eclipse i80 compound and Nikon SMZ1500 dissecting microscopes, with photomicrographs captured as outlined in Erasmus et al. (2023). Microscopes were equipped with camera lucida attachments, and pencil drawings were produced from temporarily mounted whole and dissected specimens, cleared in lactophenol and stained with lignin pink. Digital taxonomic illustrations were rendered using Adobe Illustrator CC v. 29.3 and Adobe Photoshop CC v. 26.3.

Detailed species descriptions were compiled using the DEscriptive Language for Taxonomy (DELTA; Dallwitz 2018) with a modified Gnathiidae character set (Erasmus et al. 2025). Terminology followed Monod (1926), Cohen and Poore (1994), and Svavarsson and Bruce (2012, 2019) for male morphology, and Svavarsson and Bruce (2019) and Watling (1989) for setal classification. The total body length (TL) was measured mid-dorsally, from the frontal margin of the cephalosome (including the processes, excluding the mandibles) to the posterior point of the pleotelson. Measurements were taken at the widest part of the appendage, and all values were rounded off to one decimal.

## ﻿Taxonomy

### ﻿Suborder Cymothoida Leach, 1818

**Superfamily Cymothooidea Leach, 1814**

**Family Gnathiidae Leach, 1814**

#### ﻿Genus Gnathia

Taxon classificationAnimaliaIsopodaGnathiidae

Leach, 1814, restricted synonymy

124A151F-FEEC-5AAA-9895-64B024216D3C

Gnathia Leach, 1814: 386–402; Monod 1926: 326–329 (part); Cohen and Poore 1994: 343–346.
Anceus Risso, 1816: 8.
Praniza Latreille, 1817: 54.
Zuphea Risso, 1826: 104.
Gnathia (Gnathia) s.s. — Monod 1926: 329 (part).
Gnathia (Perignathia) — Monod 1926: 554–555 (not Perignathia Monod, 1922).

#### ﻿ Gnathia spongicola

Taxon classificationAnimaliaIsopodaGnathiidae

Barnard, 1920

F21E1031-84CE-578B-98ED-ADBB201DA5A3

Figs 2, 3, 4, 5

Gnathia spongicola Barnard, 1920: 332–334, pl. XV, fig. 9.

##### Material examined.

***Lectotype*** [designated here]. South Africa • 1 ♂ (4.9 mm); Table Mountain; 33°3.348'S, 18°1.686'E; depth 347 m; 3 April 1902; SS *Pieter Fauer*; trawl; hexactinellid sponges (SAMC A099274).

***Paralectotype*.** South Africa • 3 ♂♂ (4.4–4.5 mm); same data as lectotype (SAM A4147) • 8 ♂♂ (3.9–5.6 mm); Cape Point; 34°34.314'S, 18°14.316'E; depth 247 m; 27 February 1902; SS *Pieter Fauer*; trawl; hexactinellid sponges (SAM A4148) • 1 ♂ (damaged); offshore of Lion’s Head; depth 238 m; 28 May 1900; SS *Pieter Fauer*; dredge sampling; hexactinellid sponges (SAM A4149).

***Other material*.** South Africa • 1 ♂ (damaged); Still Bay; 35°22.002'S, 22°31.002'E; depth 200 m; 20 June 1972; identified by Kensley (SAM A14603).

##### Redescription of adult male.

**Figure 2.**
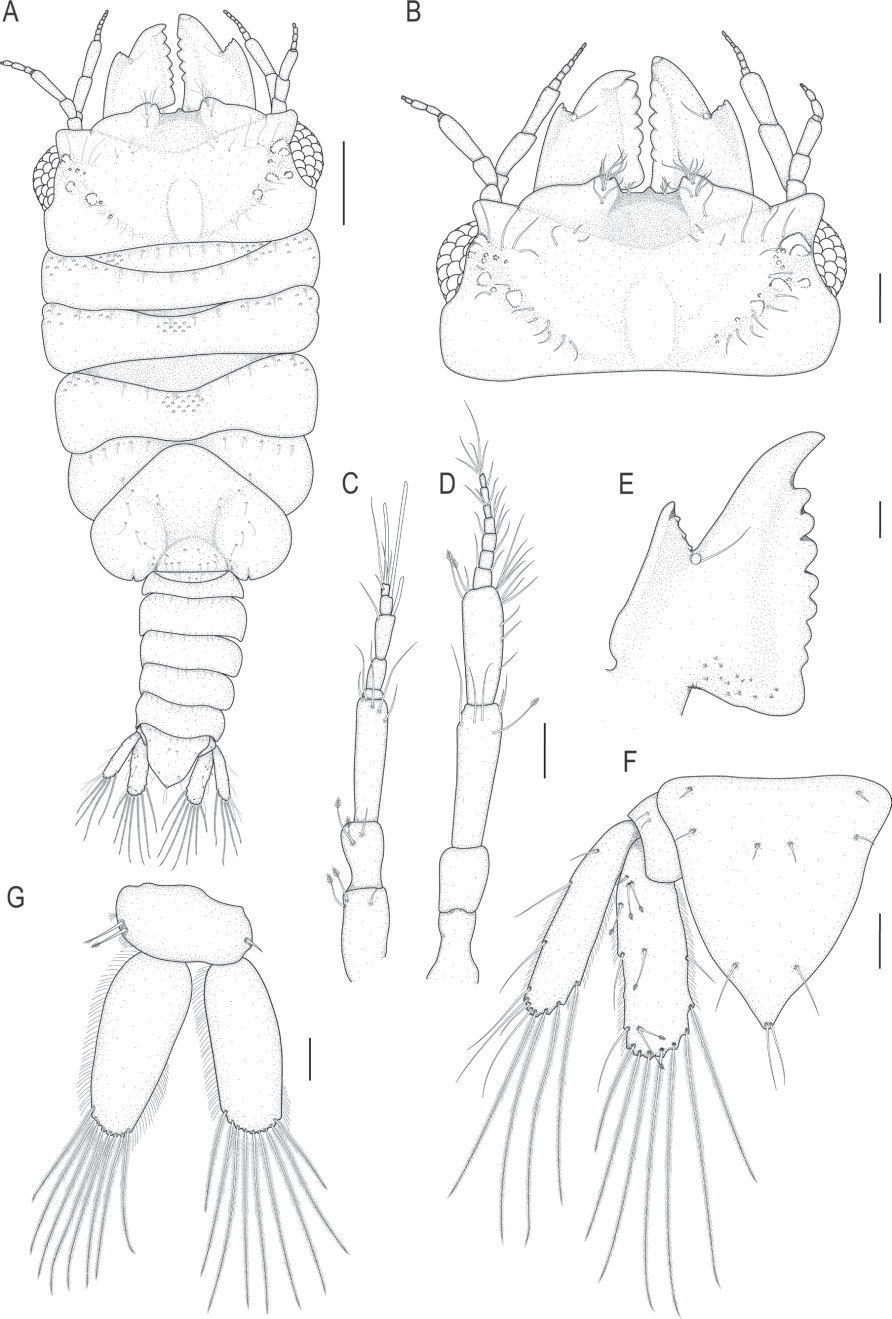
*Gnathia spongicola* (Barnard, 1920). A. Male lectotype (SAMC A099274), dorsal view of habitus; B–G. Male paralectotype (SAM A4148); B. Dorsal view of cephalosome; C. Dorsal view of antennula; D. Dorsal view of antenna; E. Dorsal view of left mandible; F. Dorsal view of pleotelson; G. Dorsal view of pleopod 2. Scale bars: 500 µm (A); 200 µm (B); 100 µm (C–E).

***Body*** (Fig. 2A) 2.5 times as long as greatest width, widest at pereonite 2 and pereonite 3; dorsal surfaces smooth, sparsely setose. ***Cephalosome*** (Figs 2B, 5A) 0.5 times as long as wide, lateral margins slightly concave anteriorly, posterior margin straight; dorsal surface with sparse granules, or tubercles around eyes; dorsal sulcus wide, deep, short; translucent region absent; para-ocular ornamentation with several tubercles and setae, posterior median tubercle present. Frontolateral processes present. Frontal margin slightly produced, median point excavated. External scissura present, narrow, shallow. Mediofrontal process present, weak, bifid, without ventral notch, with fine setae. Superior frontolateral process (Fig. 5B) present, single, strong, equally apically bifid, with 8 pairs of long simple setae. Inferior frontolateral process absent. Mesioventral margin straight; setose; anterior tip not dorsally visible. Supraocular lobe pronounced, pointed, accessory supraocular lobe not pronounced. Eyes present, round, 0.4 times as long as cephalosome length, bulbous, standing out from head surface, ommatidia arranged in rows.

***Pereon*** lateral margins narrowing posteriorly, without setae. Pereonite 1 not fused dorsally with cephalosome; dorsolateral margins fully obscured by cephalosome. Pereonite 2 wider than pereonite 1. Pereonite 4 without anterior constriction, median groove absent. Areae laterales present on pereonite 5; dorsal sulcus obscured by pereonite 6. Pereonite 6 with weak lobi laterales; lobuii weak, globular. Pereonite 7 short, narrow, and overlapping pleonite 1. ***Pleon*** covered in pectinate scales and epimera not dorsally visible on all pleonites. Pleonite lateral margins with 3 pairs of simple setae, with 1 pair of simple setae medially.

***Pleotelson*** (Fig. 2F) 1.1 times as long as anterior width, covered in pectinate scales; lateral margins smooth, anterolateral margins weakly concave, with 2 pairs of submarginal setae; posterolateral margin distally weakly concave, with 1 pair of submarginal setae; mid-dorsal surface with 1 pair of sub-median setae, apex with 2 setae.

***Antennula*** (Fig. 2C) shorter than antenna. Peduncle article 1 without tubercles, article 2 0.8 times as long as article 1; article 3 1.9 times as long as article 2; article 3 4 times as long as wide. Flagellum as long as article 3, with 5 articles; articles 3 and 4 with 1 aesthetasc, and 1 simple seta; article 5 terminating with 1 aesthetasc, and 4 simple setae. ***Antenna*** (Fig. 2D) peduncle with 4 articles; article 3 3.4 times as long as wide, 2.2 times as long as article 2, with 1 penicillate seta, and 5 simple setae; article 4 0.8 times as long as article 3, with 2 penicillate setae, 2.9 times as long as wide, and with 10 simple setae. Flagellum 0.9 times as long as article 4, 0.8 times as long as article 3, with 7 articles, terminating with 5 simple setae.

***Mandibl*e** (Fig. 2E) 1.4 times as long as width, 0.7 times as long as length of cephalosome, triangular, strongly curved distally; apex 21.3% total length; mandibular seta present. Carina present, smooth, along proximal half. Incisor elevated, standing clear of surface, distal denticulation present. Blade present, dentate, straight, along 78.7% of margin. Internal lobe absent. Dorsal lobe absent. Basal neck short. Erisma and lamina dentata absent.

**Figure 3.**
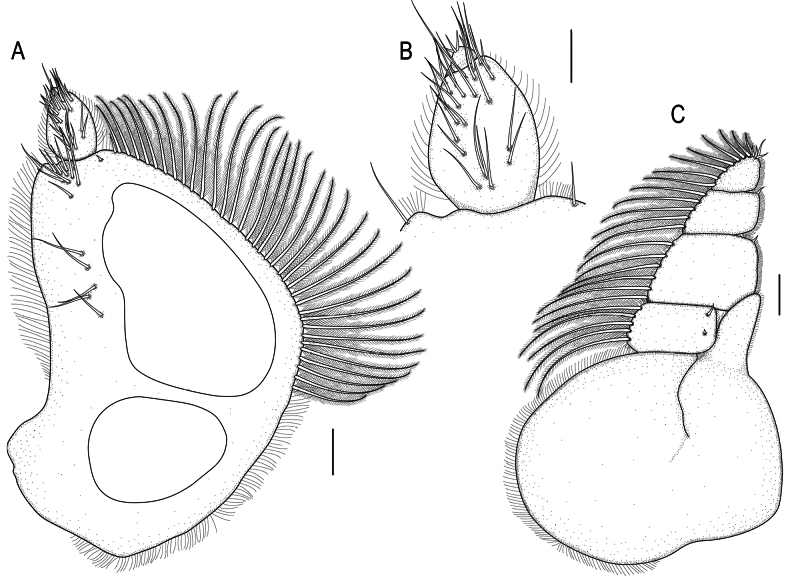
*Gnathia spongicola* (Barnard, 1920) male paralectotype (SAM A4148). A. Pylopod; B. Articles 2 and 3 of pylopod; C. Maxilliped. Scale bars: 100 µm (A, C); 50 µm (B).

***Pylopod*** (Fig. 3A) article 1 1.6 times as long as wide, with two distinct areolae, without distolateral lobe, posterior and lateral margins forming rounded curve, lateral margin with 34 large plumose setae, mesial margin with continuous scale-setae, 5 simple setae present on the surface, distal margin with 13 simple setae; article 2 1.4 times as long as wide, with 25 simple setae; article 3 minute (Fig. 3B) and fused to article 2, with 4 setae.

***Maxilliped*** (Figs 3C, 5D) 5-articled; article 1 lateral margin with continuous marginal scale-setae; article 2 lateral margin with 5 plumose setae; article 3 lateral margin with 6 plumose setae; article 4 lateral margin with 5 plumose setae; article 5 with 8 plumose setae and 4–6 simple setae; endite extending to mid-margin of article 3.

**Figure 4.**
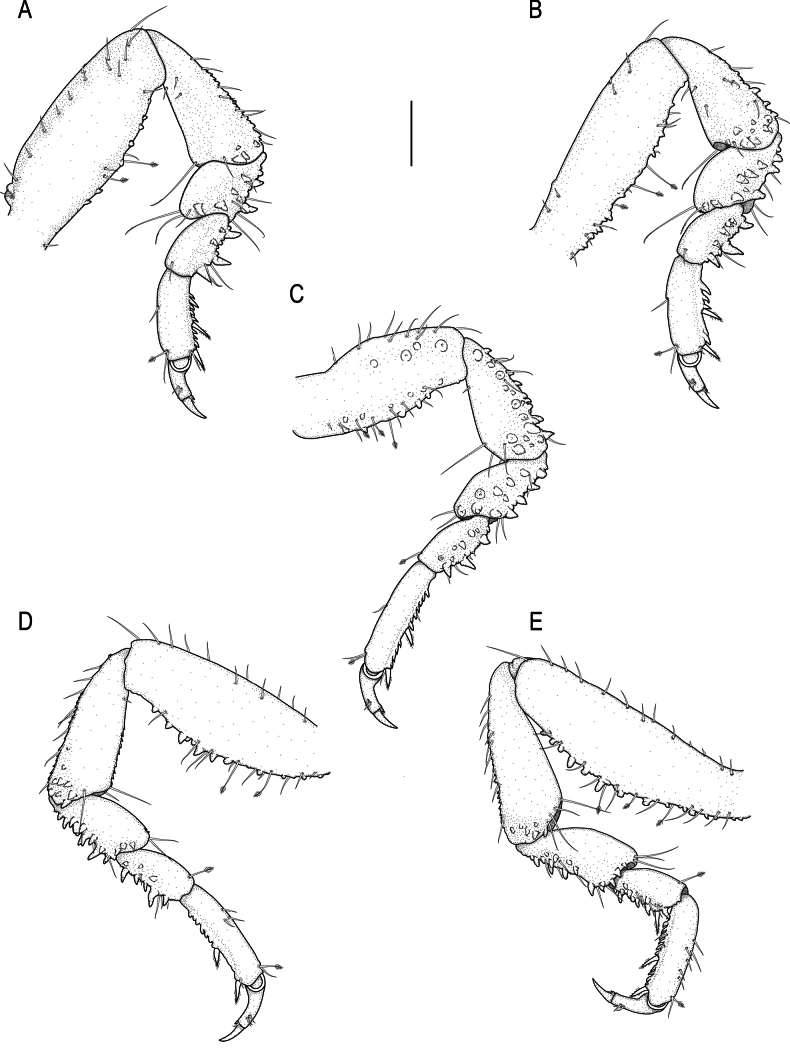
*Gnathia spongicola* (Barnard, 1920) male paralectotype (SAM A4148). A–E. Pereopods 2–6, respectively. Scale bar: 200 µm.

**Figure 5.**
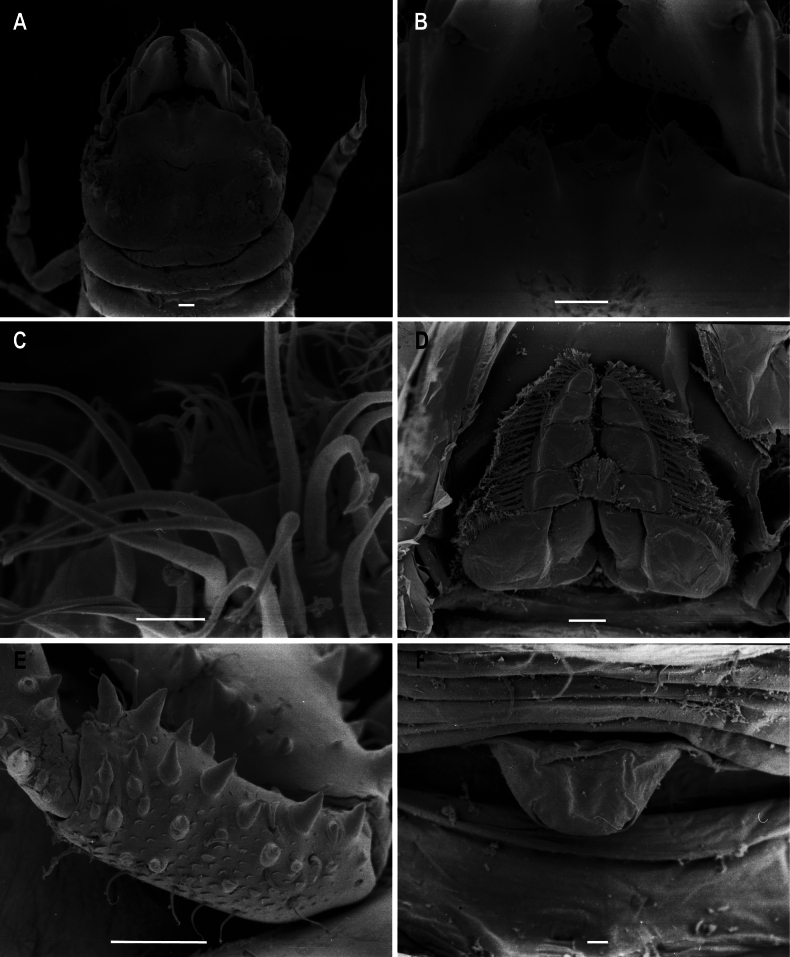
*Gnathia spongicola* scanning electron microscopy images of the male. A. Dorsal view of cephalosome; B. Dorsal view of the mediofrontal process and frontal processes; C. Article 3 of pylopod; D. Ventral view of maxilliped; E. Tubercles on pereopod 4; F. Ventral view of penial process. Scale bars: 100 µm (A, B, D, E); 10 µm (C, F).

***Pereopods 2–6*** (Fig. 4A–E) randomly covered in pectinate scales; propodus distal robust seta as long as proximal robust seta; inferior margins with prominent tubercles. Pereopod 2 (Fig. 4A) with tubercles on inferior margin of ischium to carpus; basis 2.5 times as long as greatest width, superior margin with 6 setae, inferior margin with 15 setae; ischium 0.6 times as long as basis, 1.9 times as long as wide, superior margin with 4 setae, inferior margin with 8 setae; merus 0.4 times as long as ischium, 0.9 times as long as wide, superior margin with 3 setae and bulbous protrusion; inferior margin with 5 setae; carpus 0.6 times as long as ischium, twice as long as wide, superior margin with 1 seta, inferior margin with 3 setae; propodus 0.7 times as long as ischium, 2.6 times as long as wide, superior margin with 2 simple setae and 1 penicillate seta, inferior margin with 2 simple setae, 2 short setae, and 2 robust setae; dactylus (with unguis) 0.8 times as long as propodus. Pereopods 3 (Fig. 4B) and 4 (Figs 4C, 5E) similar to pereopod 2; pereopod 5 (Fig. 4D) similar to pereopod 6 (Fig. 4E). Pereopod 6 with tubercles on basis to carpus; basis 3.2 times as long as greatest width, superior margin with 10 simple setae and 3 penicillate setae, inferior margin with 13 setae; ischium 0.7 times as long as basis, 2.8 times as long as greatest width, superior margin with 4 setae, inferior margin with 9 setae; merus 0.5 times as long as ischium, 1.8 times as long as wide, superior margin with 3 setae, inferior margin with 6 setae, without dense patch of scale-setae; carpus 0.4 times as long as ischium, 1.9 times as long as wide, superior margin with 1 seta, inferior margin with 4 setae; propodus 0.7 as long as ischium, 4.1 times as long as wide, superior margin with 8 setae, inferior margin with 1 simple seta, and 2 robust setae; dactylus (with unguis) 0.6 times as long as propodus.

***Penial process*** (Fig. 5F) 0.5 times as long as basal width, slightly produced lobe.

***Pleopod 2 exopod*** (Fig. 2G) 2.5 times as long as wide, distally narrowly rounded, medial margin weakly oblique, with 9 plumose setae; endopod 2.2 times as long as wide, distally broadly rounded, with 8 plumose setae; appendix masculina absent; peduncle 1.8 times as wide as long, mesial margin with 2 coupling setae, lateral margin with 1 simple seta.

***Uropod*** (Fig. 2G) rami extending beyond pleotelson, apices broadly rounded. Peduncle with 2 dorsal setae. Endopod 3.2 times as long as greatest width, dorsally with 5 setae; lateral margin straight, with 4 simple setae; distomesial margin sinuate, with 7 long plumose setae. Exopod not extending to end of endopod, 4.2 times as long as greatest width; lateral margin straight, 7 simple setae distolaterally; distomesial margin sinuate, with 4 long plumose setae.

##### Remarks.

*Gnathia spongicola* can be identified by several key morphological features: a slightly produced frontal margin; a weak and bifid mediofrontal process; single, strong superior frontolateral processes that are equally apically bifid; pointed and pronounced supraocular lobes; mandibles that are strongly curved distally with dentated blades; and a dorsal sulcus on pereonite 5 that is obscured by the overlapping pereonite 6.

Barnard (1920) did not designate a holotype in the original description of *G. spongicola* but provided detailed observations on the specimens examined. These syntypes are housed at the SAM, and one male specimen from this series has been designated as the lectotype and is herein redescribed. This lectotype designation is crucial to stabilise the taxonomic identity of *G. spongicola*, especially in relation to specimens previously labelled as *G. spongicola* or G. spongicola var. minor (see species descriptions below).

Among the six *Gnathia* species previously recorded from the Temperate Southern African (TSA) marine realm, *G. spongicola* most closely resembles *Gnathia disjuncta* Barnard, 1920, particularly in the division of pereonite 5 by a triangular pereonite 6. However, *G. spongicola* can be distinguished from *G. disjuncta* by its broader dorsal sulcus on the anterior cephalosome (narrow in *G. disjuncta*), the presence of 3–5 tubercles around the eye (compared to two large tubercles along the eye margin in *G. disjuncta*), and more bulbous eyes (less prominent in *G. disjuncta*). Since the original description of *G. disjuncta* in 1920, the taxonomic standards for gnathiid species description have become more refined, rendering the current description outdated. A redescription of *G. disjuncta* is therefore recommended. However, it is worth noting that the type material for *G. disjuncta* is currently missing from its vial (SAM A4152) in the SAM collection (N.J. Smit personal observation).

Outside the TSA, the division of pereonite 5 by pereonite 6 is uncommon within males of the genus *Gnathia*. *Gnathia arabica* Schotte, 1995, which exhibits partial division of pereonite 5 by pereonite 6 and prominent spines and tubercles on the pereopods, most closely resembles *G. spongicola*. However, it can be distinguished by differences in mandible shape, extensive pitting on the cephalosome, and the presence of an appendix masculina on pleopod 2. Although from a different genus, *Elaphognathia bifurcill* (Holdich & Harrison, 1980) also shows division of pereonite 5 by pereonite 6, indicating that this character is not exclusive to males of the genus *Gnathia* (Holdich and Harrison 1980).

#### ﻿ Gnathia brevicula sp. nov.

Taxon classificationAnimaliaIsopodaGnathiidae

D3E613DB-A035-568A-AED5-3DA6CDAF2671

https://zoobank.org/6CC8D0E8-EB56-42A2-8FB7-5EEE4C11E915

Figs 6, 7, 8, 9

Gnathia spongicola var. minor — Barnard 1920: 334.
Gnathia barnardi — Smit 1997: 128–142 (nomen nudum).

##### Material examined.

***Holotype*.** South Africa • 1 ♂ (2.9 mm); False Bay; 34°19.002'S, 18°33'E; depth 55 m; 4 May 1898; SS *Pieter Fauer*; trawl; branching sponges (SAMC A099275).

***Paratypes*.** South Africa • 162 ♂♂ (3.0–3.1 mm); same data as holotype (SAM A4150) • 135 ♂♂ (2.5–2.8 mm); False Bay; no coordinates provided; depth 55 m; trawl (SAM A4151).

***Other material*.** South Africa • 1 ♂ (3.2 mm); Cape Point; 34°31.3'S,18°341'E; depth 71 m; 22 May 1961; RV *Africana II*; dredge (SAM A43146) • 1 ♂ (damaged); False Bay; 34°19.002'S, 18°33'E; depth 77 m; 27 October 1961; RV *Africana II*; dredge (SAM A43147) • 1 ♂ (3.0 mm); Cape Point; 34°31.3'S, 18°31.1'E; depth 66 m; 22 May 1961; RV *Africana II*; dredge (SAM A43162).

##### Description of adult male.

**Figure 6.**
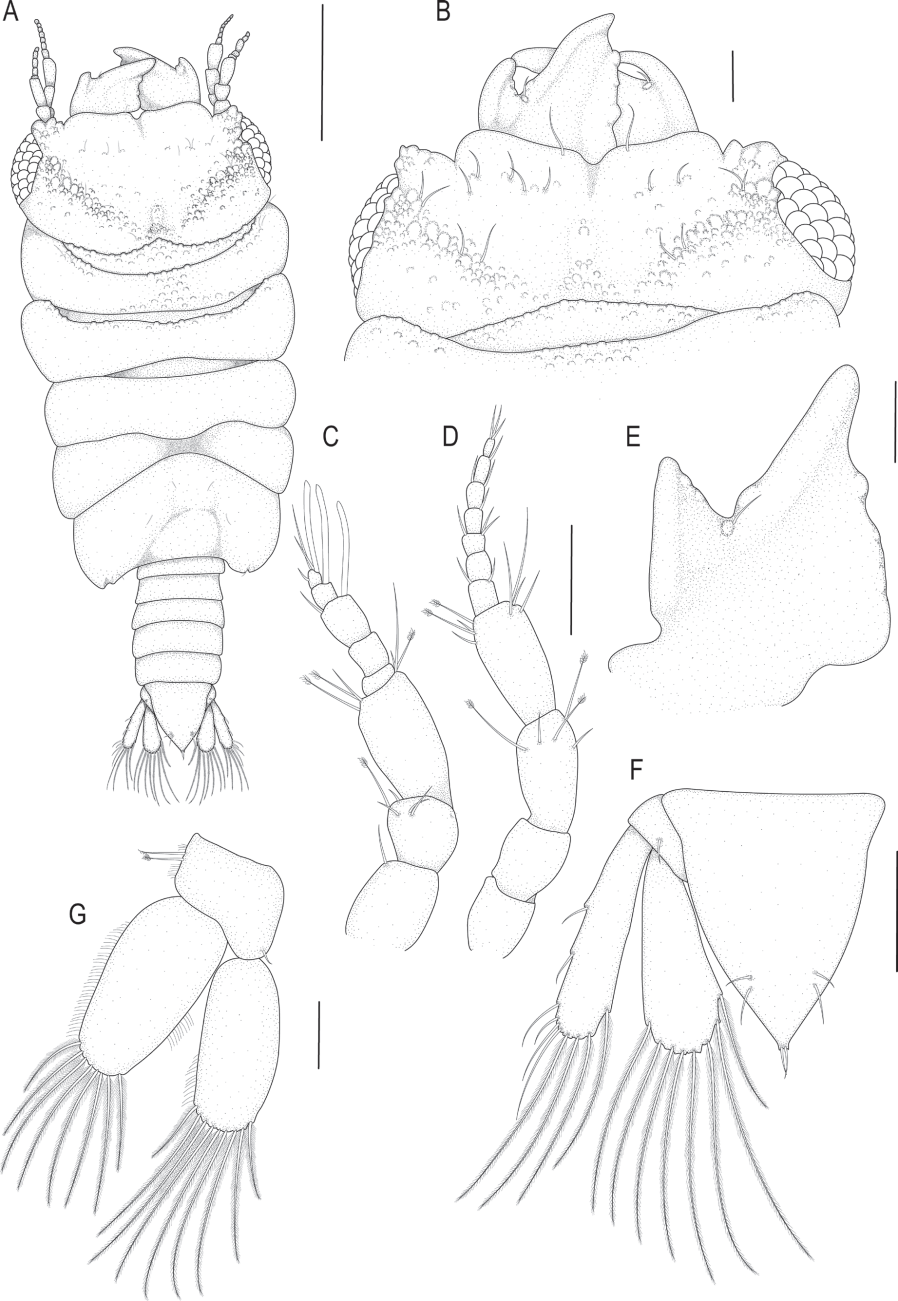
*Gnathia brevicula* sp. nov. A. Male holotype (SAMC A099275), dorsal view of habitus; B–G. Paratype (SAM A4150); B. Dorsal view of cephalosome; C. Dorsal view of antennula; D. Dorsal view of antenna; E. Dorsal view of left mandible; F. Dorsal view of pleotelson; G. Dorsal view of pleopod 2. Scale bars: 500 µm (A); 100 µm (B–G).

***Body*** (Fig. 6A) 2.3 times as long as greatest width, widest at pereonite 3; dorsal surfaces anteriorly with tubercules and posterior smooth, sparsely setose. ***Cephalosome*** (Figs 6B, 9A) 0.6 times as long as wide; lateral margins sub-parallel; posterior margin concave; dorsal surface with numerous granules; dorsal sulcus narrow, shallow, short; translucent region absent; para-ocular ornamentation strongly developed with several tubercles and setae; posterior median tubercle present. Frontolateral processes absent. Frontal margin slightly produced, median point excavate. External scissura present, narrow, deep. Mediofrontal process absent. Superior frontolateral process present, single, strong, rounded and extended, with 1 pair of long simple setae. Inferior frontolateral process absent. Mesioventral margin straight, setose, anterior tip not dorsally visible. Supraocular lobe not pronounced, wide and rounded, accessory supraocular lobe not pronounced. Eyes present, elongate, 0.5 times as long as cephalosome length, contiguous with head surface, ommatidia arranged in rows.

***Pereon*** lateral margins narrowing posteriorly, without setae; anteriorly with large granules. Pereonite 1 not fused dorsally with cephalosome; dorsolateral margins fully obscured by cephalosome. Pereonite 2 wider than pereonite 1. Pereonite 4 without anterior constriction, median groove absent. Areae laterales present on pereonite 5; dorsal sulcus obscured by pereonite 6. Pereonite 6 with weak lobi laterales; lobuii weak, globular. Pereonite 7 not visible in dorsal view. ***Pleon*** epimera not dorsally visible on all pleonites.

***Pleotelson*** (Fig. 6F) 1.1 times as long as anterior width; lateral margins smooth; anterolateral margins weakly concave; posterolateral margin straight with 2 pairs of submarginal setae; apex with 2 setae.

***Antennula*** (Fig. 6C) shorter than antenna. Peduncle article 1 without tubercles; article 2 0.8 times as long as article 1; article 3 1.9 times as long as article 2, 1.9 times as long as wide. Flagellum as long as article 3, with 5 articles; article 3 with 1 aesthetasc and 1 simple seta; article 4 with 1 aesthetasc; article 5 terminating with 1 aesthetasc and 3 simple setae. ***Antenna*** (Fig. 6D) peduncle with 4 articles; article 3 1.9 times as long as wide, 1.6 times as long as article 2, with 3 penicillate setae and 2 simple setae; article 4 as long as article 3, with 2 penicillate setae, twice as long as wide, with 6 simple setae. Flagellum with 7 articles; 1.5 times as long as article 4; terminating with 3 simple setae.

***Mandibl*e** (Fig. 6E) 1.3 times as long as width, 0.4 times as long as length of cephalosome, weakly curved distally; apex 35.4% total length; mandibular seta present. ***Carina*** present, smooth along proximal half. Incisor elevated, standing clear of surface, distal denticulation present. Blade present, weakly convex, dentate along 64.6% of margin. Internal lobe absent. Dorsal lobe absent. Basal neck short. Erisma and lamina dentata absent.

**Figure 7.**
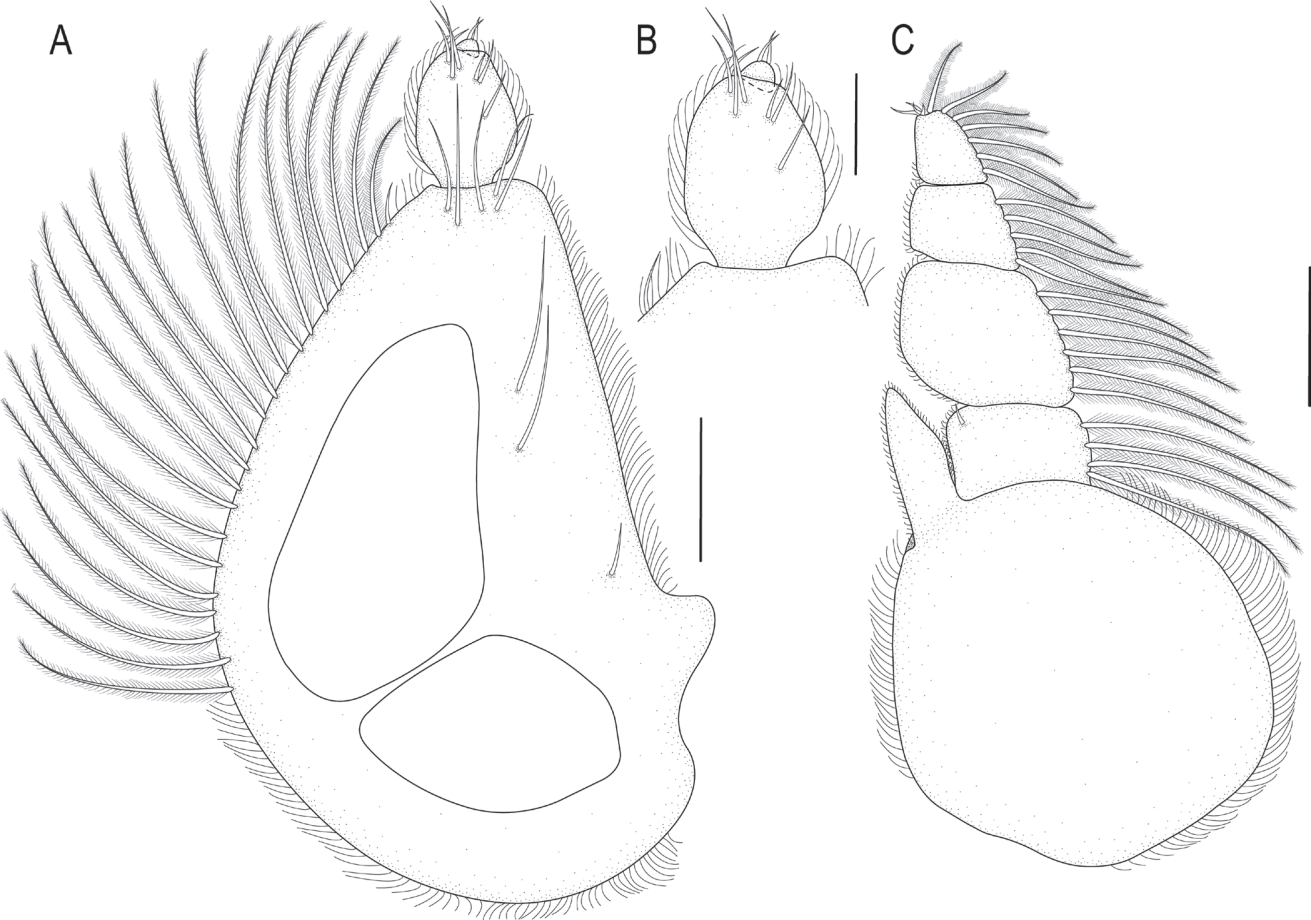
*Gnathia brevicula* sp. nov. male paratype (SAM A4150). A. Pylopod; B. Articles 2 and 3 of pylopod; C. Maxilliped. Scale bars: 100 µm (A, C); 50 µm (B).

***Pylopod*** (Fig. 7A) article 1 1.4 times as long as wide; with 2 distinct areolae; without distolateral lobe; posterior and lateral margins forming rounded curve; lateral margin with 22 large plumose setae; mesial margin with continuous scale-setae; 3 simple setae present on the surface; distal margin with 5 simple setae; article 2 1.3 times as long as wide, with 6 simple setae; article 3 minute (Fig. 7B), with 2 setae.

***Maxilliped*** (Fig. 7C) 5-articled; article 1 lateral margin with continuous marginal scale-setae; article 2 lateral margin with 4 plumose setae; article 3 lateral margin with 6 plumose setae; article 4 lateral margin with 5 plumose setae; article 5 with 7 plumose setae and 4 simple setae; endite extending to mid-margin of article 3.

**Figure 8.**
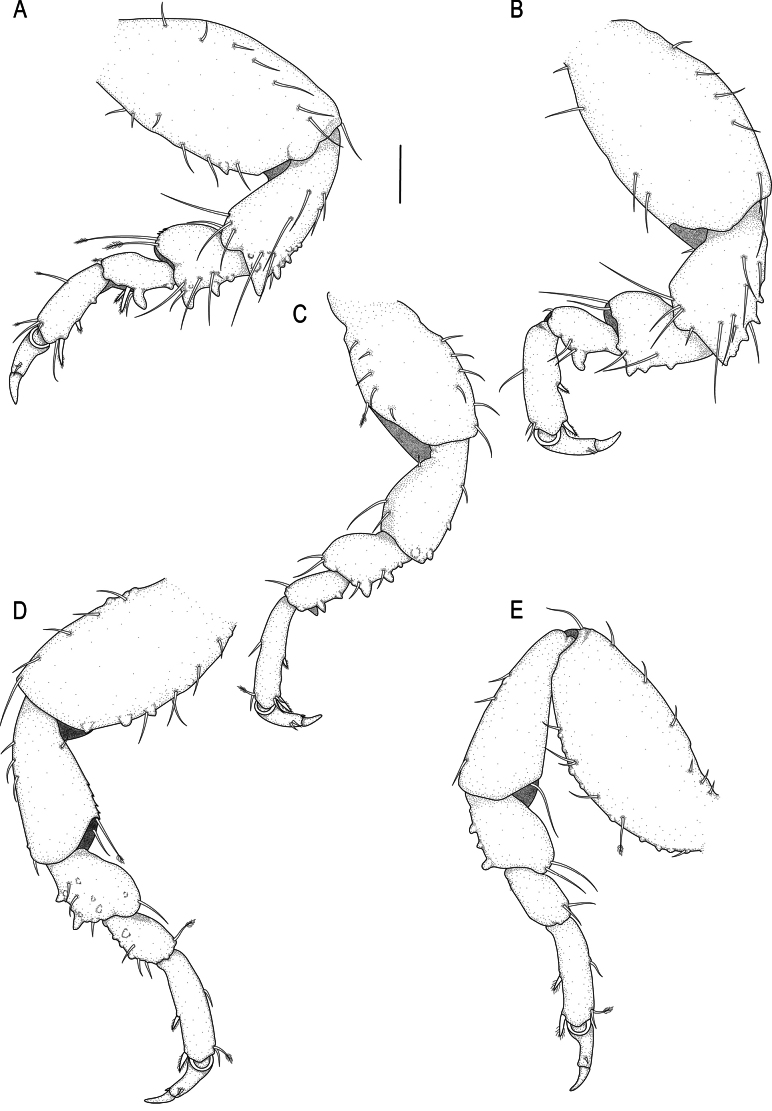
*Gnathia brevicula* sp. nov. male paratype (SAM A4150). A–E. Pereopods 2–6, respectively. Scale bar: 100 µm.

***Pereopods 2–6*** (Fig. 8A–E) with long, simple setae and randomly covered in pectinate scales; propodus distal robust seta slightly longer than proximal robust seta; inferior margins with prominent tubercles. Pereopod 2 (Fig. 8A) with tubercles on basis to carpus; basis 1.8 times as long as greatest width, superior margin with 6 setae, inferior margin with 8 setae; ischium 0.5 times as long as basis, 1.6 times as long as wide, superior margin with 4 setae, inferior margin with 7 setae; merus 0.6 times as long as ischium, as long as wide, superior margin with 2 setae; superior margin with bulbous protrusion; inferior margin with 5 setae; carpus 0.6 times as long as ischium, twice as long as wide, superior margin with 1 seta, inferior margin with 3 setae; propodus 0.8 times as long as ischium, 2.8 times as long as wide, superior margin with 2 simple setae, superior margin with 2 penicillate setae, inferior margin with 1 simple seta, and 2 robust setae; dactylus (with unguis) 1.2 times as long as propodus. Pereopods 3 (Fig. 8B) and 4 (Fig. 8C) similar to pereopod 2. Pereopod 6 (Fig. 8E) with tubercles on superior margin of basis and with tubercles on inferior margin of merus; basis 1.6 times as long as greatest width, superior margin with 5 simple setae, and 1 penicillate seta, inferior margin with 7 setae; ischium 0.8 times as long as basis, 2.1 times as long as greatest width, superior margin with 1 seta, inferior margin with 3 setae; merus 0.5 times as long as ischium, 1.2 times as long as wide, superior margin with 2 setae, without dense patch of scale-setae; carpus 0.4 times as long as ischium, 1.7 times as long as wide, superior margin with 2 setae; propodus 0.7 times as long as ischium, 3.4 times as long as wide, superior margin with 3 setae, and 2 robust setae; dactylus (with unguis) 0.7 times as long as propodus.

**Figure 9.**
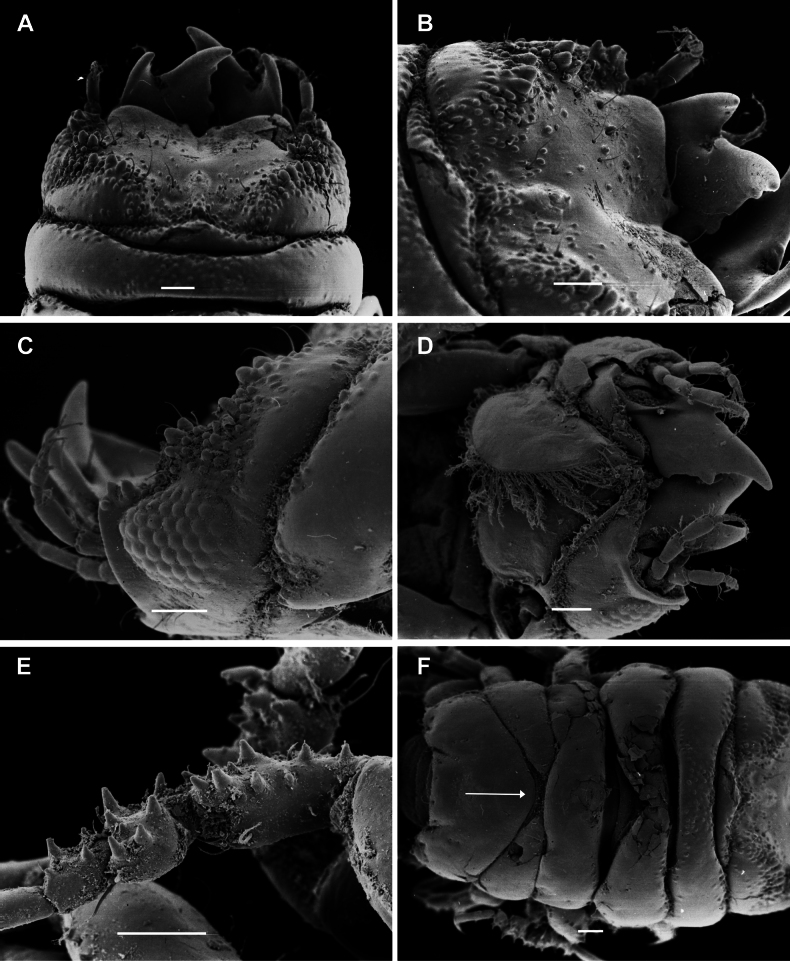
*Gnathia brevicula* sp. nov. scanning electron microscopy images of the male. A. Dorsal view of cephalosome; B. Dorsally angled view of frontal margin and mandibles; C. Ventral view of cephalosome; D. Ventral view of maxilliped; E. Tubercles on pereopod 4; F. Dorsal view of pereon, with pereonite 6 dividing pereonite 5 (arrow). Scale bars: 100 µm.

***Penial process*** 0.6 times as long as basal width, slightly produced lobe.

***Pleopod 2*** (Fig. 6G) ***exopod*** 2.1 as long as wide, distally broadly rounded, with 9 plumose setae; endopod twice as long as wide, distally broadly rounded, with 8 plumose setae; appendix masculina absent; peduncle 1.2 as wide as long, mesial margin with 2 coupling setae, lateral margin with 1 simple seta.

***Uropod*** (Fig. 6G) rami extending to pleotelson apex, apices broadly rounded. Peduncle with 1 dorsal seta. Endopod 2.6 as long as greatest width; lateral margin straight; distomesial margin sinuate, with 8 long plumose setae. Exopod extending to end of endopod, 3.8 as long as greatest width; lateral margin weakly sinuate, with 6 simple setae; distomesial margin sinuate, with 4 long, plumose setae.

##### Etymology.

The species name *brevicula* is derived from the Latin adjective *brevis*, meaning “short”, combined with the diminutive suffix -*cula*, which conveys smallness. The name thus translates as “the little short one,” in reference to the species’ smaller body size compared to *G. spongicola*. This etymology proves particularly fitting as Barnard originally regarded the specimens as a diminutive form of *G. spongicola*.

##### Remarks.

*Gnathia brevicula* sp. nov. can be distinguished by a slightly produced frontal margin; two strong frontolateral processes that are rounded and extended; wide and rounded supraocular lobes that are minimally developed; mandibles that are weakly curved distally; and a pereonite 5 divided into two by pereonite 6.

Barnard (1920) originally designated this taxon as Gnathia spongicola var. minor, considering the morphological differences too minor to justify recognition as a separate species. He attributed these variations to habitat differences, with *G. brevicula* sp. nov. inhabiting smaller, branching sponges, while *G. spongicola* was found in larger sponges. Morphological differences noted by Barnard (1920) included a smaller body size, more robust antennae, a greater number of tubercles on the cephalosome, and stouter pereopods.

Upon closer examination, however, these morphological distinctions support the recognition of *G. brevicula* sp. nov. as a separate species. In addition to Barnard’s observations, the shape of the frontal margin differs between *G. brevicula* sp. nov. and *G. spongicola*. Although both species have maxillipeds composed of five articles, the distal four articles of *G. spongicola* bear plumose setae in the sequence 5–6–5–8, while in *G. brevicula* sp. nov. the sequence is 4–6–5–7. Moreover, *G. spongicola* displays a greater number of simple setae on the pylopod than *G. brevicula* sp. nov.

The name G. spongicola var. minor was first proposed as a separate species in Smit’s (1997) dissertation under the provisional name *Gnathia barnardi*. Since then, *G. barnardi* has appeared in online sources, often cited as *G. barnardi* Smit & Basson, 2002. However, this name was never formally described following International Commission on Zoological Nomenclature (ICZN) rules and is therefore not considered a validly established species name.

#### ﻿ Gnathia lancifera sp. nov.

Taxon classificationAnimaliaIsopodaGnathiidae

250B898D-DEE2-5297-99A6-DB31F930164B

https://zoobank.org/C93615D6-3F06-471E-B685-DA7DC02C11ED

Figs 10, 11, 12, 13

Not Gnathia spongicola — Smit 1997: 98–112

##### Material examined.

***Holotype*.** South Africa • 1 ♂ (4.1 mm); east of Lake St Lucia; 27°59.5'S, 32°40.8'E; depth 550 m; 22 May 1976; RV *Meiring Naude*; dredge (SAMC A099276).

***Paratype*.** South Africa • 1 ♂ (damaged); with the same data as holotype (SAM A19326).

##### Description of adult male.

**Figure 10.**
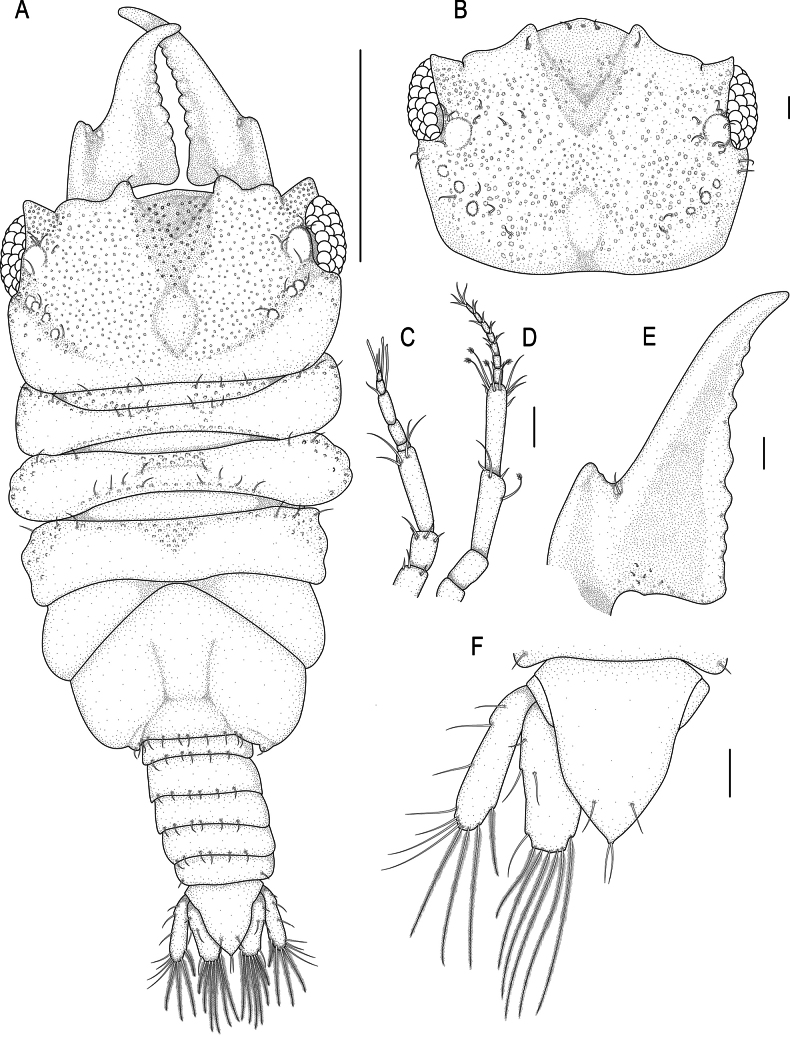
*Gnathia lancifera* sp. nov. A. Male holotype (SAMC A099276), dorsal view of habitus; B–F. Paratype (SAM A19326); B. Dorsal view of cephalosome; C. Dorsal view of antennula; D. Dorsal view of antenna; E. Dorsal view of left mandible; F. Dorsal view of pleotelson. Scale bars: 1 mm (A); 100 µm (B–E).

***Body*** (Fig. 10A) 2.3 times as long as greatest width, widest at pereonite 3; dorsal surfaces anteriorly with tubercules, sparsely setose. ***Cephalosome*** (Fig. 10B) 0.8 times as long as wide; lateral margins narrowing posteriorly; posterior margin slightly concave; dorsal surface with numerous granules; dorsal sulcus wide, deep, extended; translucent region absent; para-ocular ornamentation weakly developed and with several tubercles and setae; posterior median tubercle present. Frontolateral processes present. Frontal margin slightly produced, median point even. External scissura present, narrow, shallow. Mediofrontal process present, weak, rounded, without ventral notch, with fine setae. Superior frontolateral process present, single, strong, conical, with 1 pair of long simple setae. Inferior frontolateral process absent. Mesioventral margin slightly curved; granulated; anterior tip not dorsally visible. Supraocular lobe pronounced, rounded; accessory supraocular lobe not pronounced. Eyes present, 0.3 times as long as cephalosome length, bulbous, standing out from head surface, ommatidia arranged in rows.

***Pereon*** lateral margins narrowing posteriorly from pereonite 3, with few setae; anteriorly with numerous fine granules. Pereonite 1 partially fused dorsally with cephalosome; dorsolateral margins fully obscured by cephalosome. Pereonite 2 wider than pereonite 1. Pereonite 4 without anterior constriction, median groove absent. Areae laterales present on pereonite 5; dorsal sulcus obscured by pereonite 6. Pereonite 6 with weak lobi laterales; lobuii weak, conical. Pereonite 7 not visible in dorsal view. ***Pleon*** covered in pectinate scales and epimera not dorsally visible on all pleonites. Pleonite lateral margins with 2 pairs of simple setae, with 1 pair of simple setae medially.

***Pleotelson*** (Fig. 10E) 1.2 as long as anterior width; lateral margins smooth, anterolateral margins weakly concave; posterolateral margin straight, with 1 pair of submarginal setae; apex with 2 setae.

***Antennula*** (Fig. 10C) shorter than antenna. Peduncle article 1 without tubercles; article 2 1.4 as long as article 1; article 3 2.2 as long as article 2, 3.8 as long as wide. Flagellum with 5 articles, as long as article 3; article 3 with 1 aesthetasc and 1 simple seta; article 4 with 1 aesthetasc seta; article 5 terminating with 1 aesthetasc and 2 simple setae. ***Antenna*** (Fig. 10D) peduncle with 4 articles; article 3 3.7 as long as wide, 2 as long as article 2, with 1 penicillate seta and 3 simple setae; article 4 1.1 as long as article 3, with 4 penicillate setae. Flagellum with 7 articles, as long as article 4, 1.1 as long as article 3, terminating with 4 simple setae.

***Mandible*** (Figs 10E, 13B) crescent-shaped, strongly curved distally; apex 18.1% total length; mandibular seta present. Carina present, smooth along proximal half. Incisor elevated, standing clear of surface, distal denticulation present. Blade present, straight, dentate along 82% of margin. Dorsal lobe absent. Basal neck short. Erisma and lamina dentata absent.

**Figure 11.**
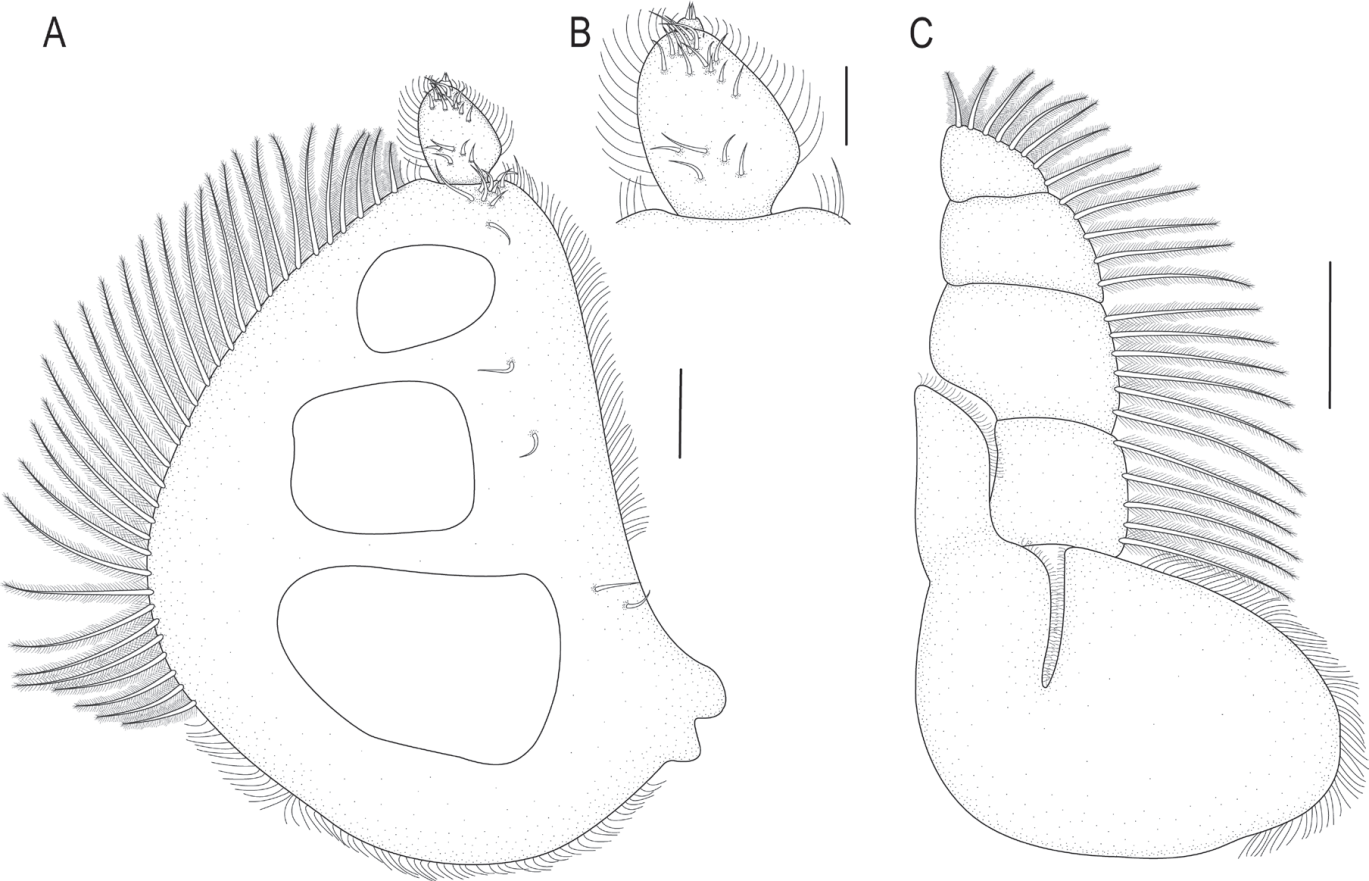
*Gnathia lancifera* sp. nov. paratypes (SAM A19326). A. Pylopod; B. Articles 2 and 3 of pylopod; C. Maxilliped. Scale bars: 100 µm (A, C); 50 µm (B).

***Pylopod*** (Fig. 11A) article 1 1.4 as long as wide, with three distinct areolae, without distolateral lobe; posterior and lateral margins forming rounded curve; lateral margin with 32 large plumose setae; mesial margin with continuous scale-setae; 4 surface simple setae present; distal margin with 8 simple setae; article 2 1.2 as long as wide, with 18 simple setae; article 3 (Fig. 11B) minute, with 4 setae.

***Maxilliped*** (Fig. 11C) 5-articled; article 1 lateral margin with continuous marginal scale-setae laterally; article 2 lateral margin with 5 plumose setae; article 3 lateral margin with 6 plumose setae; article 4 lateral margin with 5 plumose setae; article 5 with 8 plumose setae; endite extending to mid-margin of article 3.

**Figure 12.**
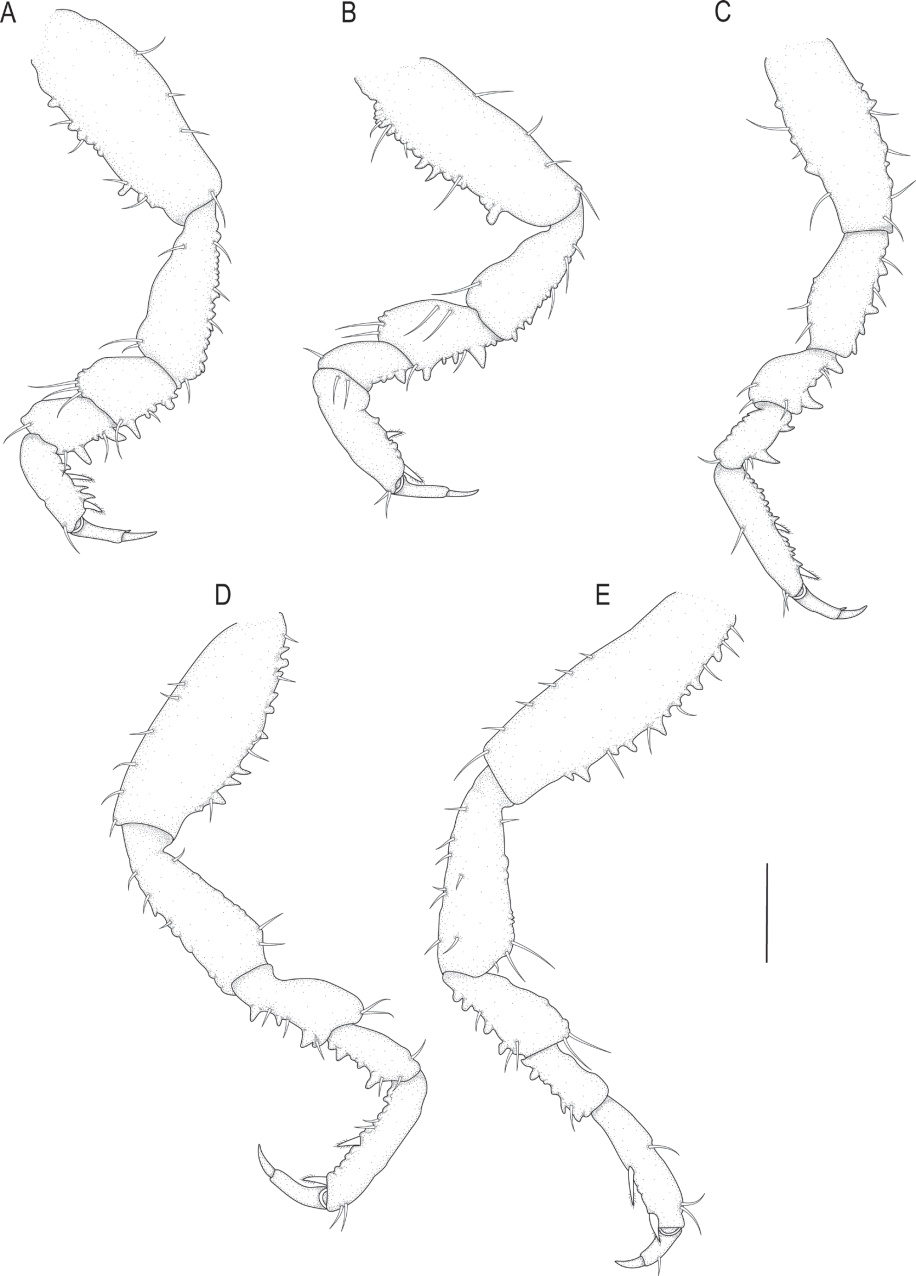
*Gnathia lancifera* sp. nov. paratype (SAM A19326). A–E. Pereopods 2–6, respectively. Scale bar: 200 µm.

**Figure 13.**
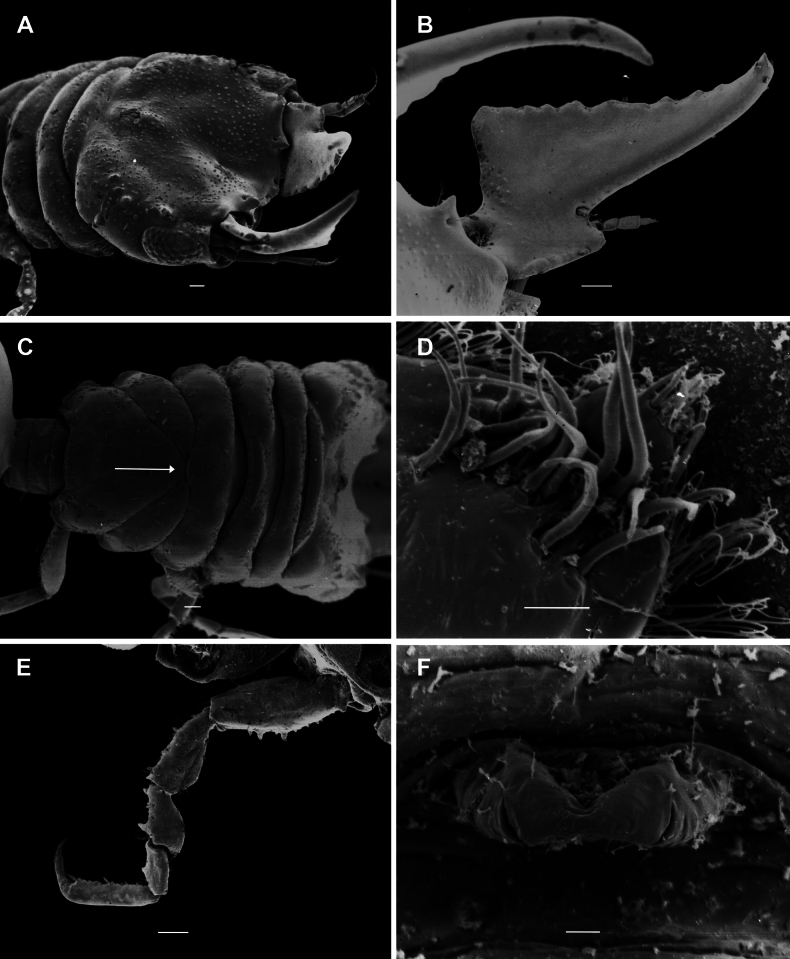
*Gnathia lancifera* sp. nov. scanning electron microscopy images of the male. A. Dorsally angled view of cephalosome; B. Right mandible, dorsal view; C. Dorsal view of pereon, with pereonite 6 dividing pereonite 5 (arrow); D. Article 3 of pylopod; E. Tubercles pereopod 4; F. Ventral view of penes. Scale bars: 100 µm (A–C, E); 10 µm (D, F).

***Pereopods 2–6*** (Fig. 12) randomly covered in pectinate scales; inferior margins with prominent tubercles. Pereopod 2 (Fig. 12A) with tubercles on basis to carpus; basis 2.2 as long as greatest width, superior margin with 4 setae, inferior margin with 4 setae; ischium 0.7 as long as basis, 2.4 as long as wide, superior margin with 3 setae, inferior margin with 4 setae; merus 0.4 as long as ischium, 0.9 as long as wide, superior margin with 3 setae, inferior margin with 2 setae; carpus 0.4 as long as ischium, 1.1 as long as wide, superior margin with 1 seta, inferior margin with 2 setae; propodus 0.6 as long as ischium, 2.7 as long as wide, superior margin with 1 simple seta and 2 robust setae; dactylus (with unguis) 0.8 as long as propodus. Pereopods 3 (Fig. 12B) and 4 (Figs 12C, 13E) mostly similar to pereopod 2; pereopod 5 (Fig. 12D) similar to pereopod 6 (Fig. 12E). Pereopod 6 with tubercles on merus and carpus and with tubercles on superior margin of basis; basis 3 as long as greatest width, superior margin with 6 simple setae, inferior margin with 6 setae; ischium 0.7 as long as basis, 2.8 as long as greatest width, superior margin with 4 setae, inferior margin with 8 setae; merus 0.5 as long as ischium, 1.8 as long as wide, superior margin with 2 setae, inferior margin with 3 setae, without dense patch of scale-setae; carpus 0.4 as long as ischium, 1.9 as long as wide, inferior margin with 1 seta; propodus 0.7 as long as ischium, 3.8 as long as wide, superior margin with 3 setae, and 2 robust setae; dactylus (with unguis) 0.5 as long as propodus.

***Penes*** medially united.

***Uropod*** rami extending beyond pleotelson apex, apices broadly rounded. Endopod 2.3 as long as greatest width, dorsally with 1 seta; lateral margin weakly sinuate, lateral margin with 3 simple setae; distomesial margin weakly convex, with 6 long plumose setae. Exopod extending to pleotelson apex, 4.3 as long as greatest width; lateral margin weakly sinuate, with 7 simple setae; distomesial margin convex, with 4 long plumose setae.

##### Etymology.

The name *lancifera* is derived from the Latin *lancea*, meaning “spear” or “lance”, and -*fera* (from *ferre*), meaning “bearing” or “carrying”. The name refers to the spear-like shape of the mandibles that resemble weapons borne by the gnathiid.

##### Remarks.

*Gnathia lancifera* sp. nov. can be recognised by the slightly produced frontal margin; a weakly rounded mediofrontal process; two strong, conical superior frontolateral processes; rounded and pronounced supraocular lobes; crescent-shaped mandibles that are strongly curved distally and dentate; and a pereonite 5 that is divided by a triangular pereonite 6.

As with *G. spongicola* and *G. brevicula* sp. nov., *G. lancifera* sp. nov. shares the uncommon feature of pereonite 5 being divided by pereonite 6. However, it can be distinguished by several key characteristics: it has significantly larger, crescent-shaped mandibles; a more prominently produced frontal margin; and relatively smaller eyes in proportion to the cephalosome. Additionally, the cephalosome of *G. lancifera* sp. nov. is densely covered with small tubercles, in contrast to the tubercle distribution in *G. spongicola* and *G. brevicula* sp. nov., where tubercles are mainly concentrated around the eyes and the posterior median region.

## ﻿Conclusion

Most of the species described from the TSA (Table 1), including those from the present study, have been recorded from three of the four ecoregions defined for the region. To date, the Namib ecoregion, located within the Benguela province, remains the only TSA ecoregion where gnathiid isopods have not yet been recorded. However, the known distribution of some TSA species extends beyond regional boundaries. For instance, *Gnathia nkulu* has been recorded off the coast of Madagascar, which lies within the Western Indo-Pacific (WIP) marine realm (Kensley et al. 2009). Similarly, *G. pantherina* was reported by Bayoumy et al. (2013) from the Arabian Gulf, also within the WIP realm. It is important to note, however, that the latter record was based solely on juvenile specimens, which are notoriously difficult to identify accurately in the absence of adult males (Smit and Davies 2004). Verification of *G. pantherina* in the WIP realm thus requires confirmation based on adult male specimens.

Among the identified species in Table 1, only three, *Gnathia africana*, *G. pantherina*, and *G. pilosus*, have complete descriptions for males, females, and juvenile life stages. Male and juvenile stages are known for *Gnathia pipinde*, while the remaining species are represented by male specimens only. Furthermore, five of the 11 species currently lack host or substrate data. These knowledge gaps represent potential areas for future research.

In conclusion, this study highlights the critical value of historical museum collections, such as those housed at the Iziko South African Museum. These collections not only reduce the need for additional specimen collection but also facilitate the reuse of existing material to redescribe species and allow the utilisation of museum collections to support the description of new species.

## Supplementary Material

XML Treatment for Gnathia

XML Treatment for Gnathia spongicola

XML Treatment for Gnathia brevicula

XML Treatment for Gnathia lancifera

## ﻿Additional information

### Use of AI

No use of AI was reported.

### Author ORCIDs

Hesmarié Botha https://orcid.org/0000-0002-4087-5499

Nico J. Smit https://orcid.org/0000-0001-7950-193X

Anja Erasmus https://orcid.org/0000-0001-8505-8583

Kerry A. Hadfield https://orcid.org/0000-0003-1308-6360

### Data availability

All of the data that support the findings of this study are available in the main text.
